# Bromodomain and extraterminal (BET) protein inhibition of IgG/IgE production in murine B cells is counter‐balanced by a strong Th2 bias

**DOI:** 10.1002/cti2.1280

**Published:** 2021-05-30

**Authors:** Zeinab Dalloul, Marie Best, Pauline Chenuet, Iman Dalloul, Sandrine Le Noir, Dieudonnée Togbé, Mylène Gador, Bernhard Ryffel, Valerie FJ Quesniaux, Yolla El Makhour, François Boyer, Jean‐Claude Aldigier, Jeanne Cook‐Moreau, Nicolas Fazilleau, Michel Cogné

**Affiliations:** ^1^ Control of the B cell Response & Lymphoproliferation CNRS UMR 7276 INSERM U1262 Limoges University Limoges France; ^2^ Infinity‐Toulouse Institute for Infectious and Inflammatory Diseases CNRS U5051, Inserm U1291 University of Toulouse III Toulouse France; ^3^ INEM ‐ UMR7355 CNRS Orléans France; ^4^ ArtImmune SAS Orléans France; ^5^ Faculty of Sciences Immunology Unit MICSU and Lebanese University Beirut Lebanon; ^6^ INSERM U 1236 University of Rennes 1 Rennes France

**Keywords:** allergic inflammation, antibody class switching, bromodomain inhibition, immune response

## Abstract

**Objectives:**

Inhibitors of bromodomain and extra terminal domain (BET) proteins are a new and growing class of anti‐cancer drugs, which decrease oncogene expression by targeting superenhancers. Antibody production is another physiological process relying on superenhancers, and it remains to be clarified whether potential immunomodulatory properties of BET inhibitors might impact humoral immunity and allergy.

**Methods:**

We thus evaluated humoral immune responses and their Th2 context *in vitro* and *in vivo* in mice following treatment with the classical BET‐inhibitor JQ1. We quantified immunoglobulin (Ig) and antibody production by B cells either stimulated *in vitro* or obtained from immunised mice. JQ1 effects on class switching and activation‐induced deaminase loading were determined, together with modifications of B, T follicular helper (Tfh) and T helper 2 (Th2) populations. JQ1 was finally tested in B‐cell‐dependent models of immune disorders.

**Results:**

Bromodomain and extra terminal domain inhibition reduced class switching, Ig expression on B cells and antibody secretion and was correlated with decreased numbers of Tfh cells. However, JQ1 strongly increased the proportion of GATA3^+^ Th2 cells and the secretion of corresponding cytokines. In a mouse allergic model of lung inflammation, JQ1 did not affect eosinophil infiltration or mucus production but enhanced Th2 cytokine production and aggravated clinical manifestations.

**Conclusion:**

Altogether, BET inhibition thus interweaves intrinsic negative effects on B cells with a parallel complex reshaping of T‐cell polarisation which can increase type 2 cytokines and eventually promote B‐cell‐dependent immunopathology. These opposite and potentially hazardous immunomodulatory effects raise concerns for clinical use of BET inhibitors in patients with immune disorders.

## Graphical abstract

While bromodomain and extra terminal domain inhibition reduces class switching, antibody secretion and T follicular helper cell number, this treatment increases the proportion of GATA3^+^ T helper 2 cells and the secretion of corresponding cytokines. In a mouse model of lung inflammation, JQ1 thus aggravates rather than improves clinical manifestations.

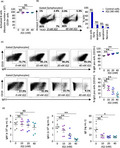

## Introduction

Inappropriate production of pro‐inflammatory class‐switched IgG or IgE is a major contributor to immunopathology. Besides direct effects, some class‐switched antibodies (Abs) also behave as natural adjuvants for T helper (Th) cells^1^, ^2^ or, in contrast, play regulatory roles such as blocking Abs of the human IgG4 class. Among immunosuppressive drugs, few can target B‐cell function and none directly modulate class switch recombination (CSR). It would thus be of strong interest to identify means to either globally depress CSR or, ideally, to specifically target the production of the most pro‐inflammatory classes such as IgE.

Class switch recombination in B cells needs recruitment of activation‐induced deaminase (AID) to initiate DNA lesions on target ‘switch’ (S) regions of the IgH locus, under control of the 3′ regulatory region (3′RR) superenhancer (SE).^3^, ^4^ This initiates double‐strand breaks within single‐stranded DNA structures and R‐loops of S regions. Chromatin readers from the bromodomain and extraterminal (BET) family proteins can promote CSR by participating in the repair of broken DNA ends^5^ but also likely impact CSR and Ig production by interacting with SEs. We thus wished to evaluate whether a BET protein inhibitor would directly impact CSR via the 3′RR SE and what would be the consequences on the humoral response.

Bromodomain and extraterminal proteins are enriched at positions of active promoters, enhancers and, to a higher extent, SE, where they promote recruitment of mediator and RNA polymerase II.^6^ SEs and BET proteins such as BRD4 contribute to inflammatory or malignant processes and activate translocated oncogenes.^7^, ^8^ Widespread development of BET inhibitors notably includes treatment of solid tumors to reduce post‐radiotherapy lung fibrosis,^8^, ^9^, ^10^, ^11^ and these drugs have rapidly entered into therapy trials, eventually ahead of science.^12^

The impact of BET inhibition on immune responses is notably poorly understood. Li *et al*.^13^ reported that the BET inhibitor JQ1 reduced Th9 responses and might be of interest in controlling airway inflammation.^13^ JQ1 also inhibited type 2 innate lymphoid cells and appeared beneficial in a short‐term (3‐day long) airway allergy model,^14^ but the impact of BET inhibition on full‐blown allergy remains to be determined.

BRD4 controls expression of Bcl‐6, the master regulator of germinal centre (GC) formation, and recruits non‐homologous end‐joining (NHEJ) factors for late DNA repair during CSR.^5^, ^15^ Except for DNA repair, no effect of BET proteins on early steps of CSR is known.^5^ Accessibility and germline transcription (GT) of target IgH switch (S) and constant (C) regions are modulated by cytokines and required for CSR.^16^

Class switch recombination needs chromatin remodelling and the occurrence of DNA loops for synapsis of S regions before their joining, which is governed by the 3′RR SE.^7^, ^8^, ^12^ The 3′RR is also targeted by locus suicide recombination (LSR).^4^ The same locus driven by the same SE can therefore be driven in opposite directions, under strong control by cytokines and T‐cell polarisation.

We wished to determine whether BET proteins might modulate humoral responses, either by directly affecting remodelling of the IgH locus or by indirectly modulating the polarity of B/T interactions. We thus evaluated the mechanisms through which a low and non‐toxic dose of JQ1 might, on the one hand, affect the B‐cell response (3′RR function, AID loading, CSR, Ig production), and, on the other hand, impact T‐cell function and the global inflammatory or allergic outcome of humoral responses *in vivo* in mice.

## Results

### Determination of the non‐toxic concentration of JQ1

JQ1 has been widely tested as an anti‐cancer agent. It proved effective against mouse tumors *in vivo*, with a maximum tolerated dose of 50 mg kg^−1^ per day, and showed anti‐proliferative effects *in vitro*. To assess its role on B cells while minimising non‐specific effects, we used low doses in the 10–40 nm range for *in vitro* assays and chose the non‐toxic dose of 30–50 mg kg^−1^ per day for *in vivo* assays.

**Figure 1:**
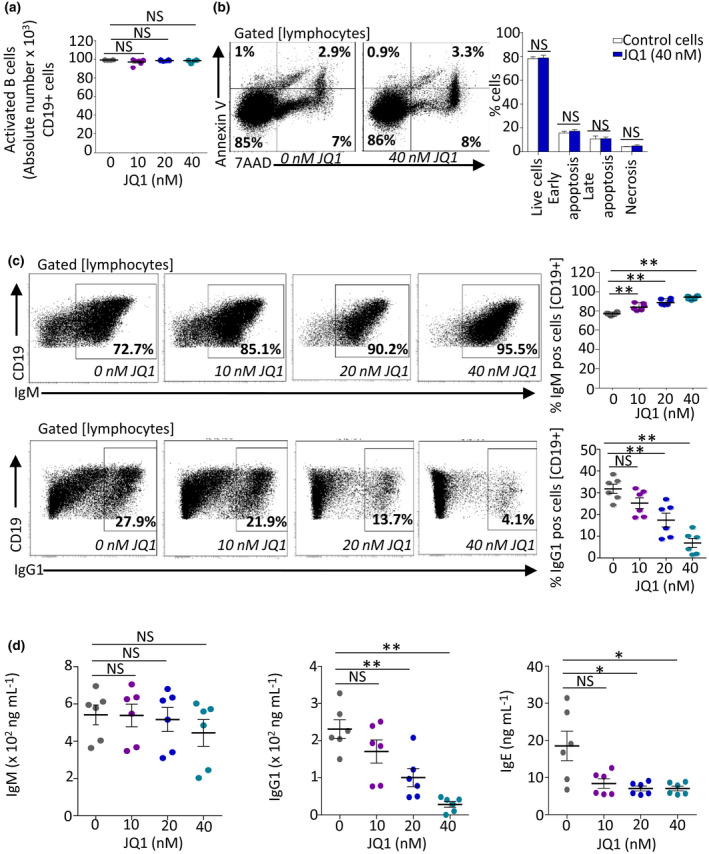
JQ1 impacts *in vitro* class switching without affecting primary B‐cell growth and viability. **(a)** Absolute numbers of B lymphocytes in day 4 LPS + IL‐4‐stimulated cultures with or without JQ1 treatment (*n* = 6 mice). **(b)** Cell death stages were analysed by staining with AnnexinV and 7AAD after 4 days culture as in **a** and in the presence of 40 nm JQ1. The *right* graph summarises the % for six mice. Cytometry gates from a representative experiment are shown (*left*). 85% represents live cells, 1% represents early apoptosis cells, 2.9% represents late apoptosis cells, and 7% represents necrotic cells. **(c)** Sorted primary B cells (95% purity) stimulated for class switching to IgG1 (with LPS + IL‐4) in the presence of 0, 10, 20 or 40 nm JQ1 were analysed for IgM and IgG1 membrane expression. Cytometry gates from a representative experiment are shown (*left*); the *right* graph summarises the % for six mice, comparing mean values. **(d)** Supernatants from *in vitro* stimulated B cells (treated 4 days with LPS + IL‐4 in the presence of 10, 20 or 40 nm JQ1) were quantified by ELISA for the production for IgM, IgG1 and IgE. Data correspond to 1 representative experiment out of 3. Values and mean % are shown for groups of six mice. NS: not significant. *P < 0.05, **P < 0.01 compared with untreated cells using the Mann–Whitney *U*‐test.

We thus validated that the low doses used *in vitro* did not significantly affect CD19^+^ B‐cell absolute numbers in LPS + IL‐4‐stimulated cultures (Figure 1a) nor influence the percentage of apoptotic cells, in cultures including up to 40 nm JQ1 (Figure 1b).

### Evaluation of *in vitro* CSR by cell cytometry and ELISA

While absolute numbers of CD19^+^ cells obtained after *in vitro* stimulation were not significantly changed in 4‐day *in vitro* stimulation cultures w/wo JQ1, we looked for qualitative variations in BCR expression and Ig secretion.

To evaluate whether JQ1 modulated class‐switching, sorted mouse B spleen cells were stimulated *in vitro* for 4 days by LPS + IL‐4 known to boost CSR and further expression of class‐switched IgG1 and IgE. Direct evaluation of class switching in B lymphocytes, by following cell‐surface BCR expression after LPS + IL‐4 stimulation, showed a strong reduction in the amount of IgG1 class‐switched cells observed, with a onefold reduction at 20 nm JQ1, a threefold reduction at 40 nm JQ1 and a reciprocal increase in IgM^+^ CD19^+^ unswitched cells (Figure 1c).

Parallel ELISA evaluation of Ig secretion in cell supernatants revealed no significant reduction in IgM levels. By contrast, and to a much stronger extent than for BCR expression, secretion of class‐switched Ig produced in such conditions (i.e. IgG1 and IgE with LPS + IL‐4) decreased for almost all doses of JQ1 tested (Figure 1d).

### AID recruitment to S regions and structure of CSR junctions

**Figure 2:**
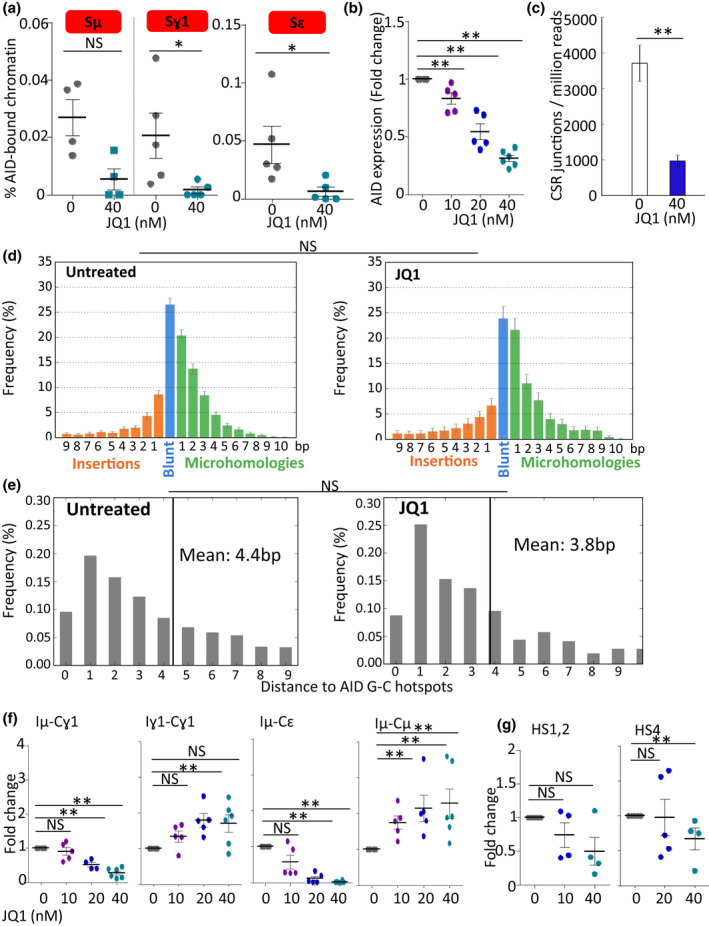
JQ1 reduces AID‐initiated CSR in primary B cells without affecting the structure of class‐switched DNA junctions. **(a)** ChIP experiments with anti‐AID Ab and qPCR quantification, showing AID recruitment to Sμ, Sɛ and Sγ1‐regions in cells stimulated with LPS + IL‐4. Data correspond to 1 representative experiment out of 2. Mean % and values are shown for groups of 4 mice. **(b)** AICDA gene expression by LPS + IL‐4‐stimulated spleen B cells treated with 10, 20 or 40 nm JQ1. Data correspond to 1 representative experiment out of 4. Mean % and values are shown for groups of five mice. **(c)** CSR junctions from stimulated primary mouse B cells were quantified by CSRseq. **(d)** Structure of junctions (one representative sample) and **(e)** relative position of breaks in Sγ1 to AID hotspots (one representative sample) analysed using CSReport. **(f)** Germline (Iγ1‐Cγ1 and Iµ‐Cμ) transcripts and pos*t‐*switch (Iμ‐Cγ1 and Iμ‐Cɛ) transcripts were analysed by RT‐qPCR in B cells stimulated by LPS + IL‐4 for 4 days in the presence of 10, 20 or 40 nm JQ1. Data correspond to 1 representative experiment out of 3. Dot plots represent fold change for groups of five mice. **(g)** Hs1,2 and hs4 transcripts were evaluated by RT‐qPCR in spleen B cells stimulated with LPS + IL‐4 for 4 days. Data correspond to 1 representative experiment out of 3. Dot plots represent means and values for groups of four mice. NS: not significant. *P < 0.05, **P < 0.01 compared with untreated cells using the Mann–Whitney *U*‐test.

We measured the loading of AID on target S regions by ChIP experiments in chromatin prepared from B cells stimulated *in vitro* for CSR (using LPS + IL‐4) and observed its drastically reduced recruitment to Sγ1 as well as Sɛ regions (Figure 2a). Part (but not all) of this strong reduction in AID loading might result from decreased expression, since a partial decrease in *Aicda* gene (encoding AID) transcription was noticed in LPS + IL‐4‐stimulated cells (Figure 2b).

Sμ‐Sγ1 CSR junctions were PCR‐amplified with specific primers, sequenced by next‐generation sequencing and analysed as previously described according to their nature and position within S regions. The most striking change was quantitative, with approximately a fourfold decrease in the number of junctions after JQ1 treatment (Figure 2c).

The repair process after the occurrence of DSBs was, however, unchanged with regard to the use of NHEJ versus the alternate microhomology‐mediated end‐joining (MMEJ) pathway; the strong predominance of NHEJ‐mediated blunt junctions, normally observed in CSR, was maintained (Figure 2d). The unaltered NHEJ/MMEJ ratio indicates that the DNA repair defect induced by JQ1 equally affects the processes of NHEJ and MMEJ. There was also no significant difference in the position of breaks, regarding their distance to the AID‐targeted RGYW/WRCY consensus motifs (Figure 2e).

While AID expression and IgH loading were decreased in the presence of JQ1, DNA breaks were thus less frequent and more focused on canonical AID target sites.

### CSR blockade occurs downstream from IgH germline transcription

To examine transcription of CSR targets in LPS/IL‐4 *in vitro* stimulated B cells, we quantified two types of IgH constant (C) gene transcripts, respectively, specific for the ‘pre‐CSR’ (Iγ1‐Cγ1 and Iμ‐Cμ germline transcripts originating from unswitched B cells) and the ‘post‐CSR’ stages (i.e. Iμ‐Cγ1 and Iμ‐Cɛ switched transcripts). Upon JQ1 treatment, we observed an increase in ‘pre‐CSR’ transcripts that are hallmarks of local IgH accessibility to CSR. By contrast, a specific decrease in IgG1 and IgE class‐switched transcripts was seen (Figure 2f).

IgH locus transcripts are not only found around coding regions and, as for other enhancers, accessibility and activity of the IgH 3′RR core enhancer elements can be assessed by evaluating their transcription into so‐called ‘eRNA’.^3^ Comparison of untreated and JQ1‐treated LPS + IL‐4‐stimulated B cells revealed a major dose‐dependent reduction in eRNA transcripts from the 3′RR hs4 and, to a lesser extent, hs1.2 core enhancer elements (Figure 2g).

JQ1 treatment thus did not merely result in globally decreased IgH transcription, but rather modulated the relative amounts of the various IgH transcripts. This indicated decreased activity of the 3′RR superenhancer. It also precisely situated the CSR blockade in between the stages of ‘pre‐CSR’ transcription (increased) and ‘post‐CSR transcription’ (decreased) and pointed to a direct alteration of the recombination step itself.

### JQ1 inhibits antigen‐specific T‐ and B‐cell humoral responses

**Figure 3:**
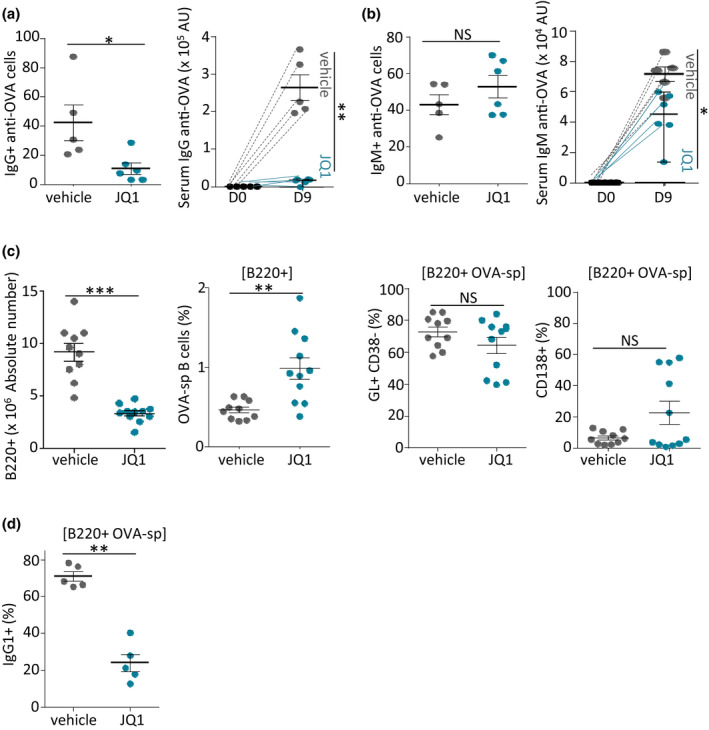
JQ1 decreases B‐cell activation *in vivo* but with less impact on Ag‐specific B cells. **(a, b)** Mice immunised with 1W1K‐OVA and receiving 50 mg kg^−1^ JQ1 daily were assayed after primary immunisation for numbers of splenocytes producing Ag‐specific IgG (a, left) or IgM (b, left) by ELISPOT and for levels of Ag‐specific Abs IgG (a, right) and IgM (b, right) by ELISA. **(c)** Absolute numbers of B220^+^ B cells (in mice treated or not with 50 mg kg^−1^ per day JQ1) were assessed in dLN (left). OVA‐specific B cells were analysed for content of germinal centre B cells (GL‐7^+^, CD38^−^ cells) and CD138^+^ plasma cells (right). **(d)** Quantification of IgG1^+^ OVA‐specific B cells. Data represent means and values for groups of 5–10 mice, from 1 out of 2 *in vivo* experiments. NS, not significant. *P < 0.05, **P < 0.01, ***P < 0.001 compared with control mice using the Mann–Whitney *U*‐test.

To evaluate *in vivo* modulation of immune responses, we immunised mice with ovalbumin (OVA), which was covalently conjugated to the T‐cell Ag model 1W1K (a variant of the Ea52‐68 immunodominant peptide from MHC class II Eα) in Alum, a Th2‐polarising adjuvant. We then followed both T and B cells of the emerging effector response in response to JQ1 treatment or control vehicle. Nine days after immunisation, draining inguinal and periaortic lymph nodes (LN) as well as spleens and sera were collected and T‐ and B‐cell primary responses were evaluated. JQ1‐treated mice showed a significant decrease (around fourfold) in specific anti‐OVA IgG secreting cells, as evaluated among sorted spleen B cells by ELISPOT (Figure 3a, left and Supplementary figure 4). Parallel serum evaluation of circulating Abs at day 9 showed a significant decrease in specific anti‐OVA IgG (Figure 3a, right and Supplementary figure 4). As a counterpart to the decreased counts of cells secreting IgG, the number of anti‐OVA‐specific unswitched (IgM‐producing) splenocytes was firmly maintained (around onefold increase, not reaching significance; Figure 3b, left and Supplementary figure 4). This was, however, associated with decreased anti‐OVA‐specific IgM detectable in sera (Figure 3b, right and Supplementary figure 4; although less markedly than for IgG), suggesting that on a ‘per‐cell’ basis, IgM secretion also tended to be lower in animals receiving JQ1.

We evaluated the extent and nature of the Ag‐specific B‐cell response (see the Methods for precise description of methodology and Supplementary figure 1 for gating strategy). Mice receiving JQ1 showed a significant decrease in total B220^+^ B cells (Figure 3c and Supplementary figure 4). OVA‐specific switched (IgD^neg^) B cells appeared higher in frequency (Figure 3c) but were lower in number in JQ1‐treated mice (data not shown), because of the overall decreased total B‐cell count.

Among OVA‐specific B cells, the frequencies of GC B cells and plasma cells did not significantly differ between both experimental groups (Figure 3c). In contrast, as observed for immunisation with OVA alone and *in vitro* in Figure 1, JQ1 impacted isotype switch, as shown by the decrease in frequency of IgG1^+^ OVA‐specific B cells (Figure 3d and Supplementary figure 4).

**Figure 4:**
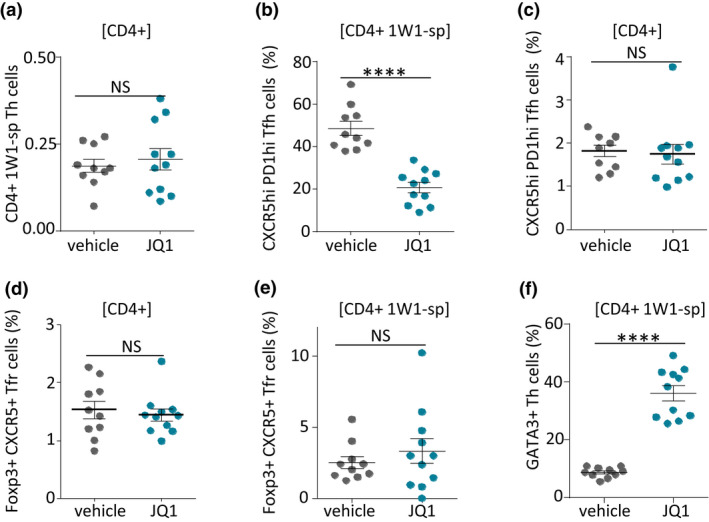
Effect of JQ1 on Ag‐specific T cells. **(a)** Frequency of 1W1K‐specific Th cells among CD4^+^ T cells in draining LN from mice 9 days after immunisation with 1W1K‐OVA in Alum and treated with vehicle or 50 mg kg^−1^ per day JQ1. **(b)** Frequency of Tfh cells (CXCR5^+^ PD1^+^) among 1W1K‐specific Th cells. **(c)** Frequency of total Tfh cells (CXCR5^+^ PD1^+^) among CD4^+^ T cells. **(d)** Frequency of total TFR cells (Foxp3^+^ CXCR5^+^) among CD4^+^ T cells, **(e)** frequency of TFR (Foxp3^+^ CXCR5^+^) among 1W1K‐specific Th cells and **(f)** frequency of GATA3^+^ cells among 1W1K‐specific Th cells. Data are from 1 out of 2 *in vivo* experiments. Means and values are shown for groups including 10 vehicle control mice and 11 JQ1‐treated mice. NS, not significant. ****P < 0.0001 compared with control mice using the Mann–Whitney *U*‐test.

In draining LN, the frequency of 1W1K‐specific T cells stained with fluorescent 1W1K‐IA^b^ tetramers (see gating strategy in Supplementary figure 2) remained similar in JQ1‐ and vehicle‐treated mice (Figure 4a and Supplementary figure 5). However, among these cells, the frequency of CXCR5^hi^ PD1^hi^ Tfh cells was significantly lower in JQ1‐treated mice (Figure 4b and Supplementary figure 5). This was not because of broad inhibition of the Tfh compartment since this decrease was observed only for the 1W1K‐specific emerging T‐cell response but not for the overall Tfh cell compartment (Figure 4c and Supplementary figure 5). Moreover, this decrease could not be accounted for by a difference in the frequency of follicular regulatory T (Tfr) cells as shown by the similar frequencies of 1W1K‐specific Tfr cells and total Tfr cells (Figure 4d and e and Supplementary figure 5). Notably, the decrease in 1W1K‐specific Tfh cells correlated with an increase in 1W1K‐specific T cells expressing GATA3, the master regulator of Th2 cells (Figure 4f and Supplementary figure 5).

Overall, these experiments further show that *in vivo* JQ1 impacts CSR intrinsically in B cells but also strongly inhibits the development of Tfh cells and enhances Th2 cell polarisation.

### JQ1 has contrasting immunomodulatory effects in an ovalbumin‐induced asthma model in mice

Using BALB/c mice which had been repetitively immunised *i.p*. with OVA, we explored whether the occurrence of allergic manifestations triggered by further administration of the immunising ovalbumin antigen could be modulated by JQ1. Four groups of mice received either saline, OVA or OVA + steroid known to prevent the occurrence of allergic asthma (Budesonide 3 mg kg^−1^, *i.n*.), or OVA + JQ1 (50 mg kg^−1^, daily *i.p* injection).

**Figure 5:**
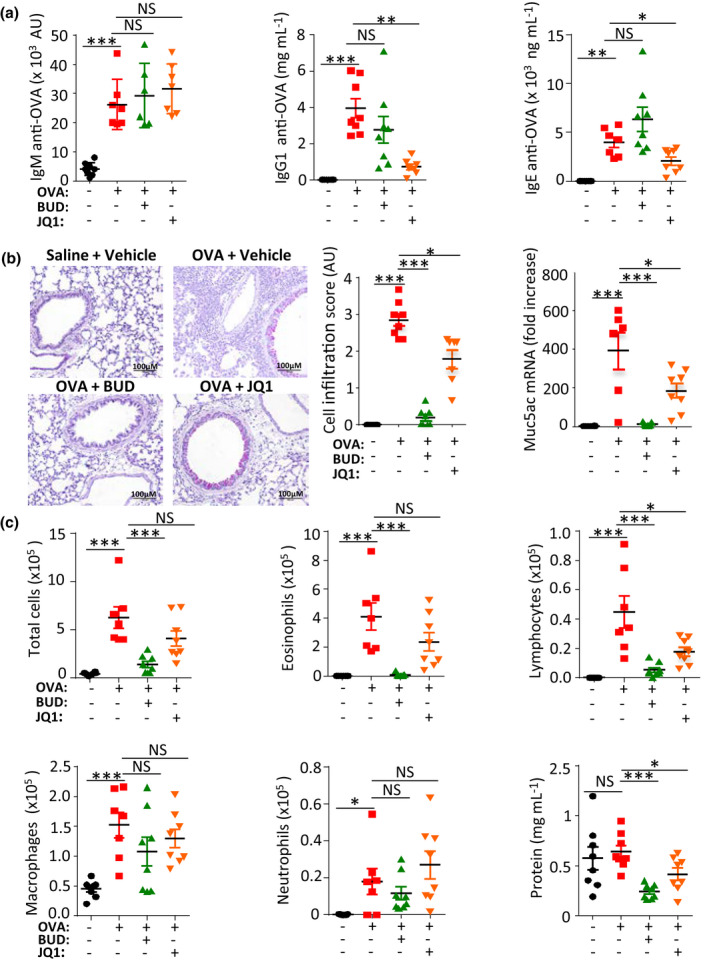
JQ1 inhibited class‐switching, Ab production and lymphocyte infiltration in an airway inflammation model. **(a)** Mice administered daily with JQ1 were assayed after repeated rounds of i.p. and i.t. immunisation by ovalbumin for levels of Ag‐specific IgM, IgG1 and IgE Abs after 25 days. **(b)** Micrograph showing haematoxylin and eosin staining of lung sections (left), inflammation scores (middle) and *Muc5ac* gene expression level (right), from mice with airway inflammation. Mice administered daily with 50 mg kg^−1^ JQ1 for 4 weeks displayed lower inflammation and less abundant cell infiltration in lungs (for each mouse, three lung sections and 10 fields for each section were analysed by two independent observers). **(c)** These mice had significantly lower counts of lymphocytes and lower protein levels in bronchoalveolar fluid (BALF). BUD: budesonide. Values are means ± standard error of the mean (SEM). Data are from 1 out of 2 *in vivo* experiments; groups include eight mice per condition tested. NS: not significant. *P < 0.05, **P < 0.01, ***P < 0.001 using the non‐parametric Kruskal–Wallis followed by Dunn's multiple comparison test.

**Figure 6:**
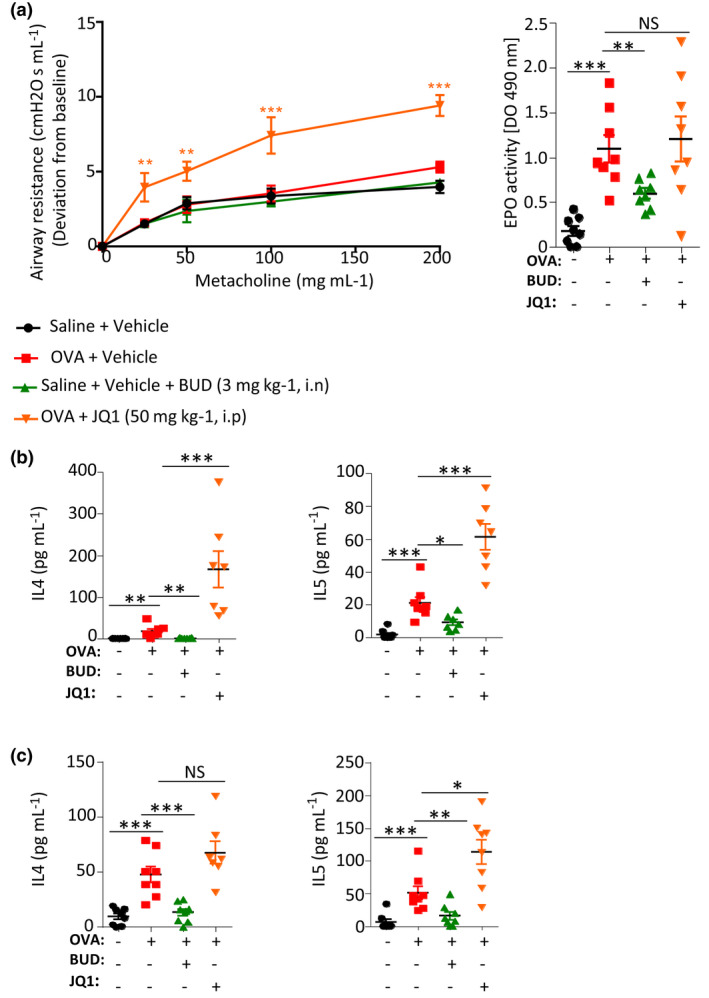
Functional defects and biased production of Th cytokines in mice with OVA‐induced lung inflammation receiving JQ1. **(a)** After repeated i.p. and i.t. OVA administration triggering allergic asthma and compared with those simply receiving OVA only, mice receiving daily steroids (but not those receiving JQ1) showed significantly lower airway resistance (a, left), and lower lung eosinophil peroxidase activity (a, right). **(b)** Mice immunised and administered daily with steroids, displayed lower levels of IL‐4 and IL‐5 in bronchoalveolar fluid (BALF) and **(c)** lower levels of IL‐4 and IL‐5 in lungs, while JQ1 had opposite effects. BUD: budesonide. Values are means ± SEM. Data are from 1 out of 2 *in vivo* experiments; groups include eight mice per condition tested. NS: not significant. *P < 0.05, **P < 0.01, ***P < 0.001 using the non‐parametric Kruskal–Wallis test followed by Dunn's multiple comparison test.

JQ1‐treated mice showed reduced systemic OVA‐specific IgG1 and IgE responses at day 25 (after primary injection of OVA followed by *i.p*. injections at days 7 and 14, and 4 *i.t*. boosters), thereby confirming a CSR defect (Figure 5a and Supplementary figure 6a). The lung tissue of OVA‐challenged WT mice showed peribronchial inflammatory cell infiltration, mucus production and hyperplasia of goblet cells as documented by semi‐quantitative scores and *Muc5ac* gene expression in the lung (Figure 5b and Supplementary figure 6b). In lungs, total cell infiltration, however, appeared only slightly reduced and less significant than in steroid‐treated mice (Figure 5b, left and middle panel, and Supplementary figure 6b). Mucus production, as evaluated by the expression of the *Muc5ac* gene in lung RNA, was also decreased by JQ1, but less significantly than by budesonide (Figure 5b, right panel and Supplementary figure 6b). Regarding bronchoalveolar fluid (BALF), the presence of lymphocytes was significantly reduced in JQ1‐treated mice, but not for other cell types (Figure 5c and Supplementary figure 6c). JQ1 also resulted in only a marginal effect on total protein levels in BALF (Figure 5c bottom right). Contrasting with the decreased production of pro‐inflammatory antibodies, JQ1 enhanced airway resistance, as measured by plethysmography (Figure 6a, left and Supplementary figure 7a), while eosinophil peroxidase activity in lungs was unaffected (Figure 6a, right and Supplementary figure 7a).

Thus, while the B‐cell response and lymphocyte infiltration were reduced by JQ1, comparable to standard steroid asthma medication (Budesonide), other aspects of lung inflammation were not. In addition and unexpectedly, the reduced Ab responses did not correlate with reduced production of type 2 cytokines. Secretion of IL‐4 and IL‐5 in BALF (Figure 6b and Supplementary figure 7b) and lungs (Figure 6c and Supplementary figure 7c) was in fact dampened only by steroids while JQ1 treatment increased Th2 cytokines in BALF. This elevated cytokine production likely explains the overall lack of functional benefit *in vivo* with JQ1. B‐cell intrinsic inhibitory effects *in vivo* (similar to those described above *in vitro*) thus seem to associate with a contrasting boost of cytokine production and Th2 polarisation.

Given this apparent dissociation of the humoral and Th2 responses, we decided to analyse the impact of JQ1 on another type of immune disorder known to be T and B cell dependent in more detail.

### Immunomodulation by JQ1 does not alleviate experimental autoimmune encephalitis

JQ1 was shown to have a beneficial effect on the severity of the classical, Th17‐dependent and non‐B‐dependent experimental autoimmune encephalomyelitis (EAE) induced with the immunodominant peptide of myelin oligodendrocyte glycoprotein (MOG).^17^ We therefore wished to study whether the immunomodulatory effects of JQ1 might be beneficial in another inflammatory condition known to be B cell dependent: EAE induced in mice by immunisation with recombinant human MOG (rhMOG).^18^ After immunisation with rhMOG, when disease onset became evident in some mice (clinical score = 1), mice were treated with vehicle or JQ1 (30 mg kg^−1^, daily *i.p* injection). In contrast to what was observed in Th17‐dependent EAE, no significant differences in the severity of B‐cell‐dependent EAE were observed between vehicle‐ and JQ1‐treated mice (Supplementary figure 3a), but disease onset, however, appeared significantly delayed (Supplementary figure 3b).

## Discussion

Class‐switched Abs are crucial actors and regulators of immune responses and can be strongly pro‐inflammatory, which is notably the case for IgE when produced in excess. Besides some direct pro‐inflammatory effects, IgG can also boost cellular responses.^1^, ^2^ Drugs targeting CSR and able to modulate the production of class‐switched Ig might thus be a grail for therapeutic immunomodulation. Since CSR is dependent upon a SE which is itself bound by BET factors, we wished to evaluate whether the BET‐inhibitor JQ1 might be active on the IgH SE which controls CSR, and whether the global effect of BET inhibition on the immune system might alleviate allergic reactions. While JQ1 and related BET inhibitors are actively studied for inhibition of tumor cell growth, they were also shown to inhibit generation of Th17‐dependent immune reactions.^7^, ^17^ By contrast, and while JQ1 is known to inhibit DNA repair during CSR in B cells,^5^ its exact impact on the whole CSR process has not been documented and its global *in vivo* impact on immune humoral responses and on B‐cell‐dependent immune disorders, such as allergic asthma, remains to be explored.

In the present study, we validated that JQ1, at doses up to 40 nm, is not cytotoxic in cultured B cells *in vitro*, and is well tolerated in mice up to a dose of 50 mg kg^−1^ per day without major adverse effects. We also confirmed a direct effect of JQ1 on CSR by following BCR expression on the cell membrane of *in vitro* stimulated B cells. This inhibition also manifested *in vivo* in immunised mice when specific Ig‐producing cells were quantified by ELISPOT, showing an increase in IgM‐producing cells *vs* a decrease in IgG‐producing cells. Consistently, an Ig secretion defect was seen *in vitro* as well as *in vivo* for class‐switched Ig.

We observed various transcriptional changes induced by BET inhibition. IgH constant gene transcripts specific for the pre‐CSR stage globally increased while switched transcripts decreased. Noticeably, expression and AID loading on S regions also clearly decreased, suggesting that not only the repair step, but also the initiation of DNA lesions was affected, contributing to the global reduction of CSR junctions observed in DNA recovered from activated B cells. The position of breaks within S regions also became more closely dependent on the proximity of AID consensus motifs and these anomalies altogether indicate a specific B‐cell maturation defect targeting the CSR step.

To better understand the overall impact of BET inhibition on the immune response, and on T cells, we immunised animals with 1W1K‐OVA, which simultaneously received 50 mg kg^−1^ per day JQ1. We noticed a decreased generation of OVA‐specific IgG‐expressing cells by FACS as well as by ELISPOT in JQ1‐treated animals, while IgM‐producing cells were unaffected or increased. These changes were in agreement with the decreased production of OVA‐specific IgG and IgE.

Furthermore, a strong decrease in 1W1K‐specific Tfh cells was noticed in the draining lymphoid organs after immunisation. JQ1 treatment, however, had an immune‐stimulatory effect on T lymphocytes, favoring Th2 cell polarisation, with increased GATA3 expression and increased production of type 2 cytokines. These data suggest that the *in vivo* decrease in CSR following BET inhibition affects not only strong B‐intrinsic modifications (as observed *in vitro*, with decreased accessibility of S regions because of decreased AID and 3′RR activity as well as decreased repair), but also exerts a complex modulation of the interactions with Tfh and Th2 cells, which are both quantitatively and qualitatively modified during immune responses.

To evaluate the potential interest of these B‐ and T‐cell changes in an Ab‐dependent immune disorder, we followed specific Ag responses in mice in the ovalbumin sensitisation protocol classically used to induce allergic airway inflammation. Importantly, objective biological markers of allergic asthma triggered by ovalbumin in immunised mice were significantly reduced in mice receiving JQ1, with a global efficiency close to that of steroids, and a notable reduction in all inflammatory cell counts in BALF. Importantly, markers from the T‐cell branch of inflammation were significantly affected in parallel, but strikingly showed increased Th2 polarisation with high IL‐4 and IL‐5 levels in BALF and lungs. Finally, JQ1 had no effect on mucus production but increased airway resistance in the allergic asthma model.

Despite a delayed disease onset, clinical signs did not improve when we explored another model of B‐cell‐dependent immune disorders: encephalomyelitis induced by rhMOG immunisation in C57Bl/6 mice. It was previously shown in this model that MOG‐specific Abs are not pathogenic, but that the disease needed antigen presentation by B cells, so that B cell‐deficient mice are resistant to rhMOG‐EAE.^18^ In the light of our observation in EAE‐induced mice, the impact of JQ1 on Tfh cell development and on specific cognate B cells was, however, not sufficient to significantly prevent the development of this B/T interaction‐dependent disease.

Altogether, both *in vivo* and *in vitro* experiments concur to demonstrate that BET proteins contribute to CSR in B cells and can be efficiently targeted by BET inhibitors. The *in vitro* CSR defect imposed by JQ1 was notably related to decrease functional interactions of AID with S regions and with the 3′RR. It translated *in vivo* by lowered numbers of cells undergoing CSR after immunisation, defective class‐switched Ig secretion and reduced development of some (but not all) biological allergy parameters.

The present study with JQ1 underlines the involvement of BET proteins at multiple levels in immune cells during humoral reactions, affecting not only B, but also T cells, which showed strongly reduced Tfh cell generation and increased Th2 cell polarisation. Since the humoral immune response relies on optimal interactions between activated GC B cells and Tfh cells, the opposing effects of JQ1 on both lineages result in a poorly predictable outcome where the apparently decreased Ab reaction is associated with identical or even more severe functional alterations. While BET proteins are often seen just as gene expression activators, it is also important to remember their dual role, since they also interact with repressive factors and BET inhibition reactivates expression of some SWI/SNF‐repressed genes.^19^ BRD4 can also directly repress some gene promoters, such as the autophagy factor LC3 (Atg8) which is activated by the JQ1 BET inhibitor.^20^

In conclusion, BET inhibitors are clearly strong immunomodulators with a broad spectrum of activity and their use in patients affected with B‐cell‐dependent/Th2‐dependent immune disorders might be hazardous. While this study required artificial Ag such as 1W1K‐OVA to precisely monitor Ag‐specific cells, our observations deserve to be validated for diverse natural antigens or autoantigens. Of note, in another unrelated model, a strong increase in Th2 cytokines was also noticed after BET inhibition in a spinal cord injury model^21^ illustrating the complex and eventually de‐repressing effects of these inhibitors. While BET inhibitors might be of interest for Th17‐dependent pathologies, their usage as class switch inhibitors will clearly have to await more specific molecules. Strategies exclusively targeting B cells and production of class‐switched immunoglobulins might notably be of interest in IgG and or IgE‐dependent immuno‐allergic conditions. In addition to our data with T cells, analysis of previous reports indicates that in different settings, BET inhibition was described in some instances as rather immunosuppressive notably by inhibiting IL‐2 responses, but shown in other instances to increase either IFN‐γ or IL‐4 production by Th2 cells, while eventually inhibiting FoxP3 expression and Treg function, thereby removing an important brake on Th2 responses.^22^, ^23^, ^24^, ^25^ Altogether and despite the strong repressing impact on intrinsic B‐cell responses, the conflicting effects on T‐cell regulation show that BET inhibition should be considered to have a potential hazardous impact on immunity and immunopathology, thus calling for extensive immune monitoring of current clinical trials in cancer patients.

## Methods

### Mice

Our research received ethical agreement APAFIS no. 16152‐2018071717143183v2. Six‐ to‐12‐week‐old BALB/c or C57BL/6 (depending upon the disease model studied) mice (maintained at 21–23°C with a 12‐h light/dark cycle) were used for our experiments.

### Cell cultures

Splenocytes were collected, red blood cells were lysed, and CD43^+^ cells were depleted using CD43 microbeads (Miltenyi Biotec, Bergisch Gladbach, Germany). B cells were cultured for 4 days (for ELISA, qPCR and flow cytometry) and for 2 days (for ChIP), in RPMI containing 10% FCS with lipopolysaccharide (LPS, 1 μg mL^−1^) + IL‐4 (40 ng mL^−1^) with or without JQ1 (Tocris, Bristol, UK). JQ1 was used at 5–40 nm.

### ELISA and ELISPOT

ELISA was performed on sera and supernatants from *in vitro* stimulated and JQ1 (10, 20 and 40 nm) treated and untreated sorted primary B cells for the detection of IgM, IgG1 and IgE secretion. Plates were coated with mAbs specific for IgM, IgG1 or IgE (Southern Biotech, Birmingham, AL, USA). Sera or supernatants were added and incubated for 2 h at 37°C. After washing, alkaline phosphatase (AP)‐conjugates of goat anti‐mouse IgM, IgG1 or IgE (Southern Biotech) were incubated 1 h at 37°C. After washing and addition of AP substrate, absorbance was measured at 405 nm.

Anti‐OVA‐specific Abs produced *in vivo* after immunisation were evaluated in sera from JQ1‐treated or untreated mice (50 mg kg^−1^). ELISA plates were coated with 10 μg mL^−1^ ovalbumin. Sera were then incubated for 2 h at 37°C, and plates were treated as above. Anti‐OVA IgG and IgM were detected with horseradish peroxidase (HRP)‐conjugated anti‐mouse IgG or IgM (Southern Biotech).

For ELISPOTs, specific IgG and IgM anti‐OVA Ab‐secreting cells were quantified using splenocytes from mice sacrificed 9 days after immunisation. Splenocytes were seeded in duplicate at a density starting at 5 × 10^5^ per well, followed by twofold serial dilutions in 96‐well MultiScreen HTS plates (Millipore, Burlington, MA, USA) coated with 200 μg per well OVA. Cells were incubated overnight at 37°C and then removed by washing with PBS/0.01% Tween. Plates were incubated for 1 h at 37°C with 1 μg per well AP‐coupled anti‐IgG or anti‐IgM. After washing, AP substrate was added. Plates were washed and dried, and images were analysed for spot numbers using Nis‐Ar software (Nikon, Tokyo, Japan).

### Flow cytometry

Class switch recombination was assessed using the following Abs: anti‐mouse IgM‐APC (Clone II/41; eBiosciences, Waltham, MA, USA), CD19‐APC H7 (Clone 1D3; BD Biosciences, San José, CA, USA), IgG1‐BV421 (Clone A85‐1; BD Biosciences), CD138‐APC (Clone 281‐2; BD Biosciences), B220‐BV421 (Clone RA3‐6B2; BD Biosciences). Apoptosis was evaluated using AnnexinV‐FITC (BD Biosciences) according to the manufacturer's instructions, and 20 µL 7AAD (BD Biosciences Pharmingen, San José, CA, USA) was added 5 min prior to analysis. Data were acquired on a Beckton Dickinson LSRII‐Fortessa cytometer and analysed with the BD FACSDiva 6.1.3 software (Becton Dickinson, Franklin Lakes, NJ, USA).

Cell suspensions were prepared in PBS/2% FCS, 5 mm EDTA. For Ag‐specific Th and B‐cell analyses, organs were dissociated, filtered, and treated with 2.4G2 for 10 min. To track Ag‐specific CD4^+^ T cells, cells were incubated with PE‐1W1K‐IAb tetramer (7 μg mL^−1^) and APC anti‐CXCR5 (REA 215, Miltenyi Biotec, 1:10) or BV421 anti‐CXCR5 (2G8; Becton Dickinson, Franklin Lakes, NJ, USA, 1:50) for 2 h at room temperature. The tetramer 1W1K‐IAb was from the NIH Tetramer Core Facility. To track Ag‐specific B cells, cells were stained for 60 min with OVA‐Alexa488 (Invitrogen, Carlsbad, CA, USA) at a final concentration of 1 μg mL^−1^ and then incubated on ice for 45 min with fluorophore labelled mAbs. The following mAbs purchased from BD Biosciences were used: anti‐CD4 (RM4‐5, 1:200), anti‐CD138 (281‐2, 1:500), anti‐CD95 (Jo2, 1:500), anti‐GL‐7 (GL‐7, 1:500), anti‐B220 (RA3‐6B2, 1:500), anti‐GATA3 (L50‐823, 1:200), anti‐IL‐4 (11B11, 1:100), anti‐IL‐10 (JES5‐16E3, 1:200), CD19‐APC‐369 H7, IgG1‐BV421, CD138‐APC, and B220‐APC. Apoptosis was evaluated using AnnexinV‐FITC (BD Biosciences) according to the manufacturer's instructions and 20 μL 7AAD (BD Pharmingen) was added 5 min prior to analysis.

The following mAbs purchased from eBioscience were used: anti‐PD1 (J43, 1:500), anti‐Foxp3 (JFK‐16s, 1:200), anti‐IFNγ (XMG1.2, 1:100), anti‐CD4 (RM4‐5, 1:1000), anti‐Tbet (4B10, 1:200), anti‐IL‐13 and anti‐CD44 (IM7, 1:800), and anti‐mouse IgM‐APC. Anti‐IgD (11‐26c, 1:1000) was from Biolegend (San Diego, CA, USA). For IFNγ, IL‐4, IL‐13 and IL‐10 intracellular staining, cell suspensions were incubated either for 4 h at 37°C in the presence of Golgi Plug (BD Bioscience), 4 µm monensin, 50 ng mL^−1^ PMA and 2 µm ionomycin, or with 1W1K (10 µg mL^−1^) overnight and with Golgi Plug for 1 h at 37°C. Then, cell suspensions were fixed and permeabilised using the Invitrogen Fixation/Permeabilization kit. Fixable Viability Dye eFluor506 (eBioscience) excluded dead cells. Data were collected on a BD LSRII‐Fortessa (BD Biosciences) and analysed using BD FACS Diva 6.1.3 and either Infinity or FlowJo (Tree Star, Becton Dickinson) software.

### Transcription analysis (RT‐qPCR)

After 4 days of *in vitro* stimulation with or without JQ1, B cells were collected and RNA was extracted to evaluate post‐switch and germline transcripts. RNA was prepared using standard techniques. cDNA was synthesised using the High Capacity cDNA Reverse Transcription kit (Thermo Fisher Scientific, Waltham, MA, USA). Iμ‐Cμ, and Iɣ1‐Cɣ1 germline transcripts and Iμ‐Cɣ1, Iμ‐Cɛ post‐switch transcripts were quantified. Quantitative PCR was performed using power SYBR green (Applied Biosystems, Foster City, CA, USA) and specific oligonucleotides listed in Supplementary table 1. These transcripts were normalised to glyceraldehyde‐3‐phosphate dehydrogenase (*Gapdh*) transcripts (reference Mm99999915‐g1). For hs1,2 and hs4 eRNA amplification, the following primers listed in Supplementary table 1 were used.

RNA extracted from lung tissue was also evaluated by RT‐qPCR/SYBR green analysis in order to measure *Muc5ac* gene expression, as a marker of mucus production. Gene expression was normalised to the expression of mouse *Hprt1*. Primers used to assess *Muc5ac* gene expression are listed in Supplementary table 1.

### Amplification of Sμ/Sγ junctions and Ion torrent next‐generation sequencing

DNA from LPS + IL‐4‐stimulated cells (treated with or without 40 nm JQ1) was extracted using the classical phenol/chloroform protocol. Sμ/Sγ junctions were amplified in triplicate by nested PCR with 100 ng DNA (Phusion HF polymerase; New England Biolabs, Ipswich, MA, USA) using the following primers listed in Supplementary table 1. Each library was prepared using 200 ng PCR2 product. Barcoded libraries with 200‐pb read lengths were prepared using Ion Xpress Plus Fragment Library Kit (Thermo Fisher Scientific) according to the manufacturer's instructions. Each barcoded library was mixed in equal amounts and diluted to 100 pm. Libraries were run on an Ion PI v3 chip on the Ion Proton sequencer (Life Technologies‐Thermo Fisher Scientific). Data analysis was performed using CSReport.^26^

### ChIP

ChIP experiments were performed on LPS + IL‐4‐stimulated CD43^−^ spleen cells incubated with or without 40 nm JQ1. Briefly, 15 × 10^6^ B cells were cross‐linked at room temperature for 15 min in 15 mL PBS with 1% formaldehyde. The reaction was quenched with 2.125 m glycine. After lysis, chromatin was sonicated to 0.5–1 kb using a Vibracell 75043 (Thermo Fisher Scientific). Following dilution in ChIP buffer (0.01% SDS, 1.1% Triton X‐100, 1.2 mm EDTA, 16.7 mm Tris/HCl, pH 8.1 and 167 mm NaCl), chromatin was precleared by rotating for 2 h at 4°C with 50 mL 50% protein A/G slurry (0.2 mg mL^−1^ sheared salmon sperm DNA, 0.5 mg mL^−1^ BSA and 50% protein A/G; Sigma, St Louis, MO, USA). Cell equivalents (1 × 10^6^) were saved as input, and the remaining cell equivalents were incubated overnight with anti‐AID rabbit polyclonal Abs (kindly provided by P. Gearhart) or control Abs. Immunoprecipitation was with protein A/G. Cross‐linking was reversed by overnight incubation (70°C) in TE buffer with 0.02% SDS and chromatin was phenol/chloroform extracted. QPCR assays used to evaluate precipitated DNA from Sμ, Sγ1 and Sɛ used the primers listed in Supplementary table 1.

### *In vivo* Th immune response in C57/Bl6 mice

C57BL/6 (CD45.2^+^) mice were purchased from Janvier (Genest Saint Isle, France). Females (8–12 weeks) were used for experimental procedures. Aluminium hydroxide adjuvant (Alum) was from Thermo Scientific. The T‐cell Ag 1W1K (EAWGALANKAVDKA) covalently coupled to OVA was bought from Genecust (Boynes, France). Mice were immunised subcutaneously (*s.c*.) at the tail base and intraperitoneally (*i.p*.) with 40 µg 1W1K‐OVA in Alum. Mice were then treated daily *i.p*. with vehicle or JQ1 (50 mg kg^−1^) for 9 days after which draining inguinal and periaortic LN as well as spleens were collected. The extent and nature of the Ag‐specific Th cell response were then evaluated by flow cytometry using fluorescent 1W1K‐IA^b^ tetramers (obtained from the NIH Tetramer Core Facility, Emory University, Atlanta, GA, USA).

### OVA‐induced allergic asthma model

BALB/c female mice at 8 weeks of age were immunised i.p. on days 0, 7 and 14 with 20 μg grade V ovalbumin (Sigma) emulsified in 2 mg Alum gel in a total volume of 200 μL. For control mice, 200 μL saline was injected. Mice were challenged at days 21–24 with 10 μg OVA by intra‐tracheal (i.t.) administration to provoke allergic asthma with analysis of the allergic response at day 25. Control mice received saline. To compare anti‐allergic therapies, mice immunised with OVA received daily either budesonide (3 mg kg^−1^, *i.n*.), JQ1 (50 mg kg^−1^, *i.p*) or vehicle (hydroxypropyl‐b‐cyclodextrin (100 mg mL^−1^), from day −1 until day 24 and 1 h before immunisation or challenge.

### Bronchoalveolar lavage and differential cell counts

Bronchoalveolar lavage (BAL) was performed by washing lungs four times with 0.5 mL saline at room temperature. After centrifugation at 400 *g* for 10 min, supernatants from the first lavage (cell‐free BAL fluid) were stored at −80°C for cytokine analysis. Cells were diluted with Turk's solution and counted and 2 × 10^5^ cells were centrifuged onto microscope slides (cytospin at 113 *g* for 10 min, at RT). Air‐dried preparations were fixed and stained with Diff‐Quik (#130832, Medion Diagnostics AG, Merz & Dade, Germany). A total of 200 cells were observed by oil immersion light microscopy to determine the relative percentage of each cell type and absolute number of the differential cell count.

### Airway resistance measurement

Mice were anaesthetised by intra‐peritoneal injection of ketamine (100 mg kg^−1^; Merial, Lyon, France) and xylasine (10 mg kg^−1^; Bayer, Leverkusen, Germany), paralysed using d‐tubocurarine (0.125%, Sigma) and intubated with an 18‐gauge catheter. Respiratory frequency was set at 140 breaths per min with a tidal volume of 0.2 mL and a positive end‐expiratory pressure of 2 mL H_2_O. Increasing concentrations of aerosolised methacholine (0, 25, 50, 100 and 200 mg mL^−1^, Sigma) were administered. Resistance was recorded with a plethysmograph (Buxco, London, UK). Baseline resistance was restored before administering the subsequent doses of methacholine.

### Lung homogenisation for evaluation of cytokines and EPO activity

After BAL, the entire lung was perfused with isotonic solution through the right heart ventricle to flush the vascular content and lungs were weighed and frozen at −20°C until use. The right lung was homogenised in PBS containing anti‐protease cocktail (# P8340; Sigma) and centrifuged, and the supernatant was aliquoted and stored at −20°C until analysis. EPO activity was determined in lung supernatants by colorimetric assay. Following centrifugation, 100 μL of supernatants was placed in a plate with 50 μL substrate solution, corresponding to 11 mL Tris HCl + 200 mm OPD pellets + 100 μL 30% H_2_O_2_). After 1‐h incubation at 37°C in a shaker, EPO activity was determined as 490 nm absorbance against medium. The right lung post‐caval lobe was removed and placed in RNAlater (Thermo Fisher Scientific) for 24 h and snap‐frozen for further analysis. The left lung was removed and preserved in 4% formaldehyde for histopathological analysis.

### Lung histology and mucus production

After BAL and lung perfusion, the left lung was fixed in 4% buffered formaldehyde (#15225582, Thermo Fisher Scientific) for at least 24 h for standard microscopic analysis. Sections (3‐μm) were stained with haematoxylin and eosin (H&E) and periodic acid–Schiff (PAS). Peribronchial infiltrates were assessed by a semi‐quantitative score (0–5) by two independent observers on three independent lung sections, and with 10 different fields analysed for each section. Inflammatory cells including eosinophil infiltration were assessed. A quantitative evaluation of mucus production in lungs was obtained in parallel by assessing *Muc5ac* gene expression using RT‐qPCR (as described above) on RNA from lung tissue.

### Cytokine measurement

IL‐4 and IL‐5 concentrations in BALF and lung homogenates were determined by Luminex immunoassay (Millipore) by using MagPix system (Bio‐Rad, Hercules, CA, USA) according to the manufacturer's instructions.

### Induction and assessment of B‐cell‐dependent experimental autoimmune encephalomyelitis

C57BL/6J mice were injected s.c. with 100 µg rhMOG (Eurogentec, Liège, Belgium) emulsified in CFA (Sigma) containing 200 µg heat‐killed mycobacterium tuberculosis (Mtb) H37RA (Merck, Darmstadt, Germany) on day 0. Additionally, mice received 200 ng Bordetella pertussis toxin i.v. (Sigma) in 0.2 mL PBS on days 0 and 2. Individual animals were observed daily, and clinical scores were assessed with a 0‐ to 5‐point scoring system (as shown in Supplementary figure 3) as follows: 0 = no clinical disease, 1 = loss of tail tone only, 2 = mild monoparesis or paraparesis, 3 = severe paraparesis, 4 = paraplegia and/or quadriparesis and 5 = moribund or death. Sick mice (scores over 1) received vehicle or JQ1 (30 mg kg^−1^, daily *i.p* injection). Moribund mice were euthanised.

### Statistics

Statistical tests were performed using GraphPad Prism (GraphPad, San Diego, CA, USA, *P < 0.05, **P < 0.01, ***P < 0.001, ****P < 0.0001). The non‐parametric Mann–Whitney *U*‐test, the Kruskal–Wallis test followed by Dunn's post‐test, two‐way ANOVA tests and Student's *t*‐tests were used when adequate, in order to determine significant differences between conditions.

## Conflict of Interest

D Togbe and P Chenuet are employees at ArtImmune. All the authors have no conflict of interest related to this study.

## Author contributions

**Zeinab Dalloul:** Investigation; Methodology; Data curation; Formal analysis; Writing‐original draft (supporting); Writing‐review & editing (supporting). **Marie Best:** Methodology; Investigation. **Pauline Chenuet:** Methodology; Investigation. **Iman Dalloul:** Methodology; Investigation. **Sandrine Le Noir:** Data curation (supporting); Investigation; Formal analysis. **Dieudonnèe Togbé:** Data curation (supporting); Writing‐original draft (supporting); Writing‐review & editing (supporting). **Mylène Gador:** Methodology; Investigation. **Bernhard Ryffel:** Conceptualization (supporting); Funding acquisition (supporting); Writing‐original draft (supporting). **Valerie FJ Quesniaux:** Conceptualization (supporting); Funding acquisition (supporting). **Yolla El Makhour:** Conceptualization (supporting). **François Boyer:** Formal analysis. **Jean‐Claude Aldigier:** Conceptualization (supporting); Funding acquisition (supporting). **Jeanne Cook‐Moreau:** Conceptualization (supporting); Formal analysis; (supporting); Writing‐original draft (supporting); Writing‐review & editing (supporting). **Nicolas Fazilleau:** Conceptualization (supporting); Data curation (supporting); Funding acquisition (supporting); Writing‐original draft (supporting); Writing‐review & editing (supporting). **Michel Cogné:** Conceptualization (lead); Data curation (lead); Formal analysis; Funding acquisition (lead); Writing‐original draft (lead); Writing‐review & editing (lead).
