# Supplementary material for: Bromodomain and extraterminal (BET) protein inhibition of IgG/IgE production in murine B cells is counter‐balanced by a strong Th2 bias
Source: Clin Transl Immunology. 2021 May 30;10(6):e1280. doi: 10.1002/cti2.1280 (PMC8164936; doi:10.1002/cti2.1280)
Supplement: Supplementary file 1 [file CTI2-10-e1280-s001.pdf]

Supplementary table 1: List of primers

Primers	Sequence 5’ -> 3’	Use
Iɣ1 forward	GGCCCTTCCAGATCTTTGAG	Transcription analysis (RT-qPCR)
Cɣ1 reverse	ATGGAGTTAGTTTGGGCAGCA	
Iμ forward	ACCTGGGAATGTATGGTTGTGGCTT	
Cμ reverse	TCTGAACCTTCAAGGATGCTCTTG	
Cε reverse	AGCGATGAATGGAGTAGC	
Hs1.2 forward	ATCAGTACCAGAAACAAGGC	
Hs1.2 reverse	TTGGGGTGAACCTGCAGC	
Hs4 forward	TTTAGTCTCAGCAAGACCC	
Hs4 reverse	AATGGGGCTTTCCACGCC	
muc5ac forward	CAGCCGAGAGGAGGGTTTGATCT	
muc5ac reverse	AGTCTCTCTCCGCTCCTCTCA	
PCR1 forward	AGAGACCTGCAGTTGAGGCC	Amplification of Sμ/Sγ junctions
PCR1 reverse	TCAGGGAARTAVCCYTTGACC AGG CA	
PCR2 forward	CCAGCCACAGTAATGACCCAG	
PCR2 reverse	CCARKGGATAGACHGATGGGG	
Sμ forward	TCTAAAATGCGCTAAACTGAGG	ChIP
Sμ reverse	AGCGTAGCATAGCTGAGCTC	
Sγ1 forward	GGGGGAGGAGATATCCAAGA	
Sγ1 reverse	CAGCTCTTTTGCAGGTCTTG	
Sε forward	CTTGACCACCGAATGTCCTT	
Sε reverse	GATTCCTCTCCAGCCTCTCC	

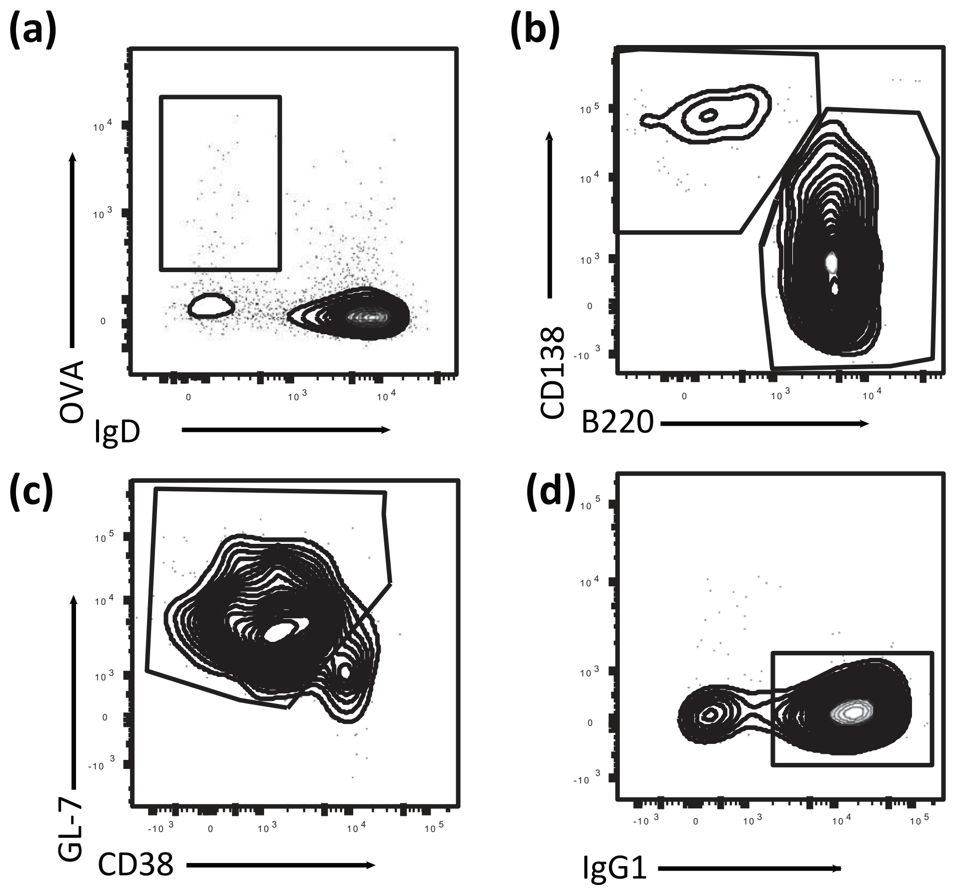

Supplementary figure 1 : Gating strategy for B lymphocytes.
After 9 days OVA immunization of C57Bl/6 mice, draining LN were collected and lymphocytes analyzed for detection of OVA-specific B cells **(a)**. **(b)** Gating of plasma ($CD138^{+}$) and B220+ B cells. Detection of GC B cells ($GL^{-}7^{+}CD38^{+}$) **(c)** and $IgG1^{+}$ $B220^{+}$ B cells **(d).**

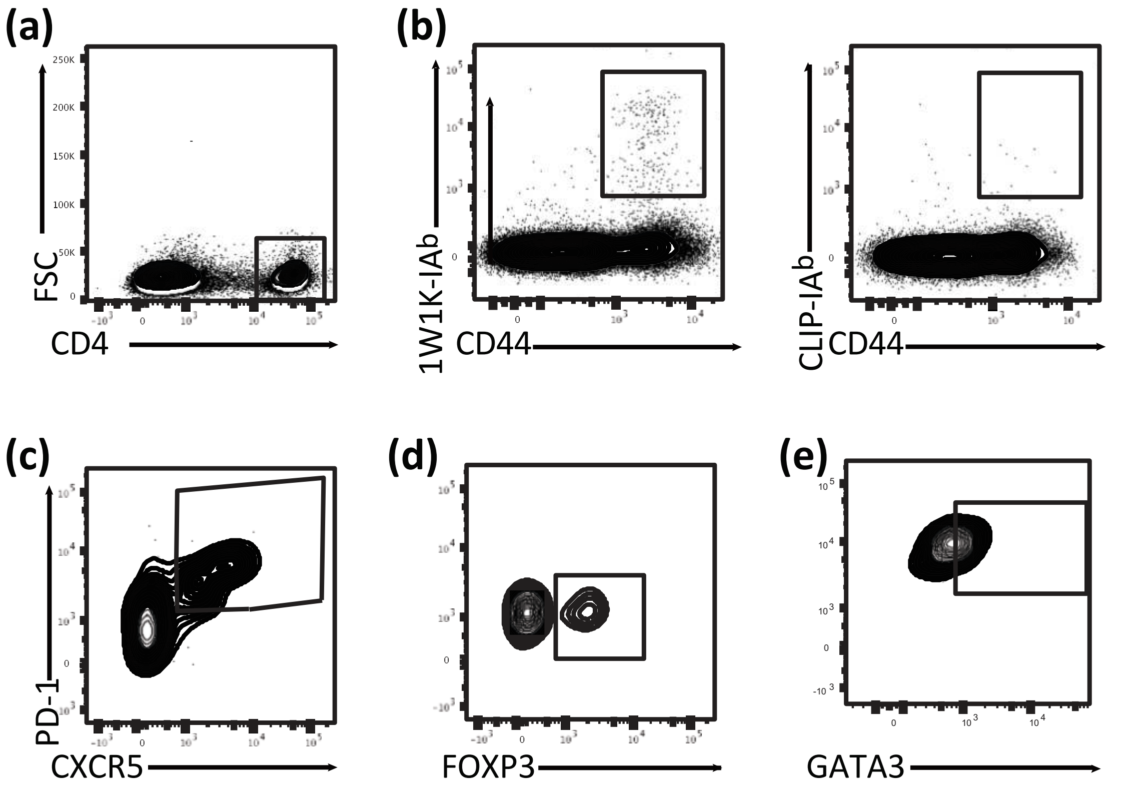

Supplementary figure 2 : Gating strategy for T lymphocytes.
After 9 days OVA immunization of C57Bl/6 mice, draining LN were collected and lymphocytes were gated for CD4+ cells **(a)**. 1W1K-specific Th cells were identified with 1W1K-IAb tetramer compared to an irrelevant CLIP-IAb tetramer **(b)**. Detection of Tfh (CXCR5^{+}PD^{-}1^{+}) **(c)**, FOXP3^{+} **(d)** and GATA3^{+} **(e)** cells.

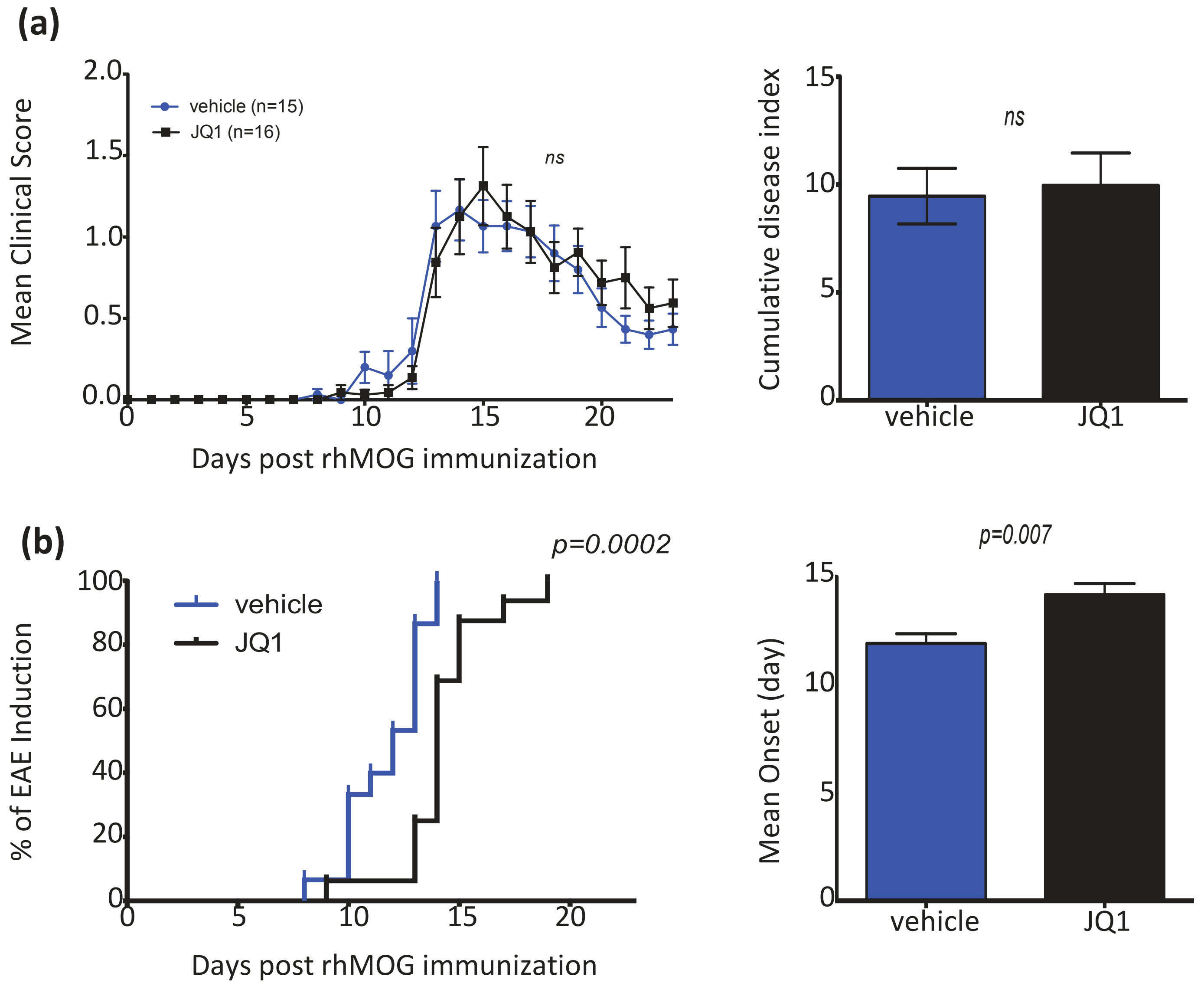

Supplementary figure 3: Effect of JQ1 on B-cell dependent EAE

EAE disease progression was monitored daily in C57/B6 mice (data shown for 1 out of 2 experiments). Treatment with DMSO (vehicle; n = 15 mice) or JQ1 (30 mg kg^{-1} ; n = 16 mice) was started day 11 after immunization and lasted for the remaining duration of the study. **(a)** Curves of clinical score progression and cumulative disease index are shown, together with **(b)** onset delay in treated mice. Data are representative of three independent experiments. Error bars represent standard deviation. Statistics were calculated using the 2-way ANOVA test for mean clinical score. Interaction accounts for approximately 0.94% of the total variance. F = 0.54. DFn=23 DFd=666. *P*- value = 0.9639. Treatment accounts for approximately <0.1% of the total variance. F = 0.24. DFn = 1, DFd = 666. *P*-value = 0.6216. The Mann-Whitney *U*-test was used for cumulative disease index and mean onset and the Log-rank (Mantel-Cox) test for EAE induction.

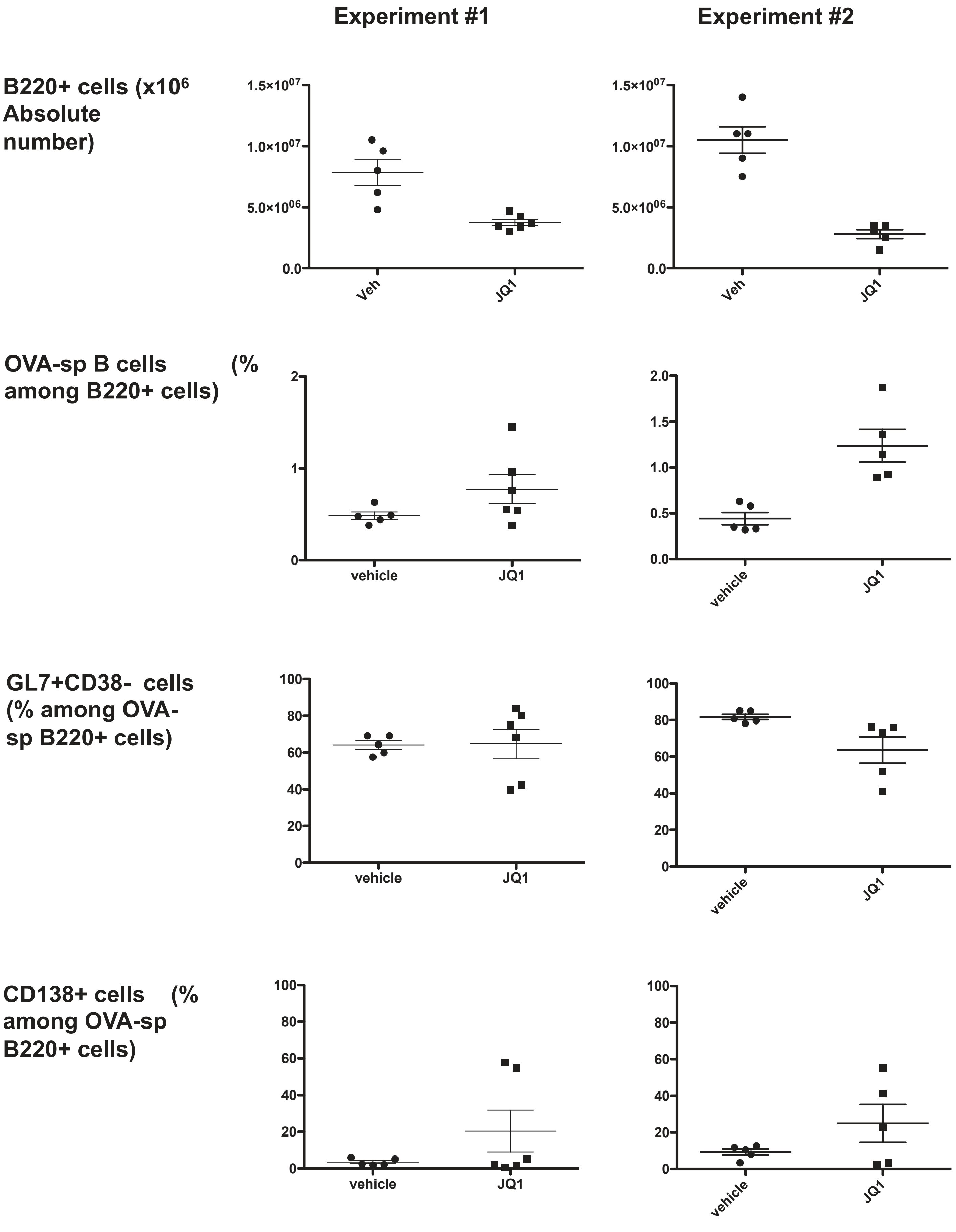

Supplementary figure 4: Replicate experiments for effects of JQ1 on B cell activation *in vivo*. Intraperitoneal and subcutaneous immunisation of C57BL/6 mice with 1W1K-OVA in Alum, with or without daily JQ1 treatment from day -1 to day 8 (Exp #1, right: n=5 for vehicle, n=6 for JQ1 / Exp #2, left: n=5 for vehicle, n=5 for JQ1)

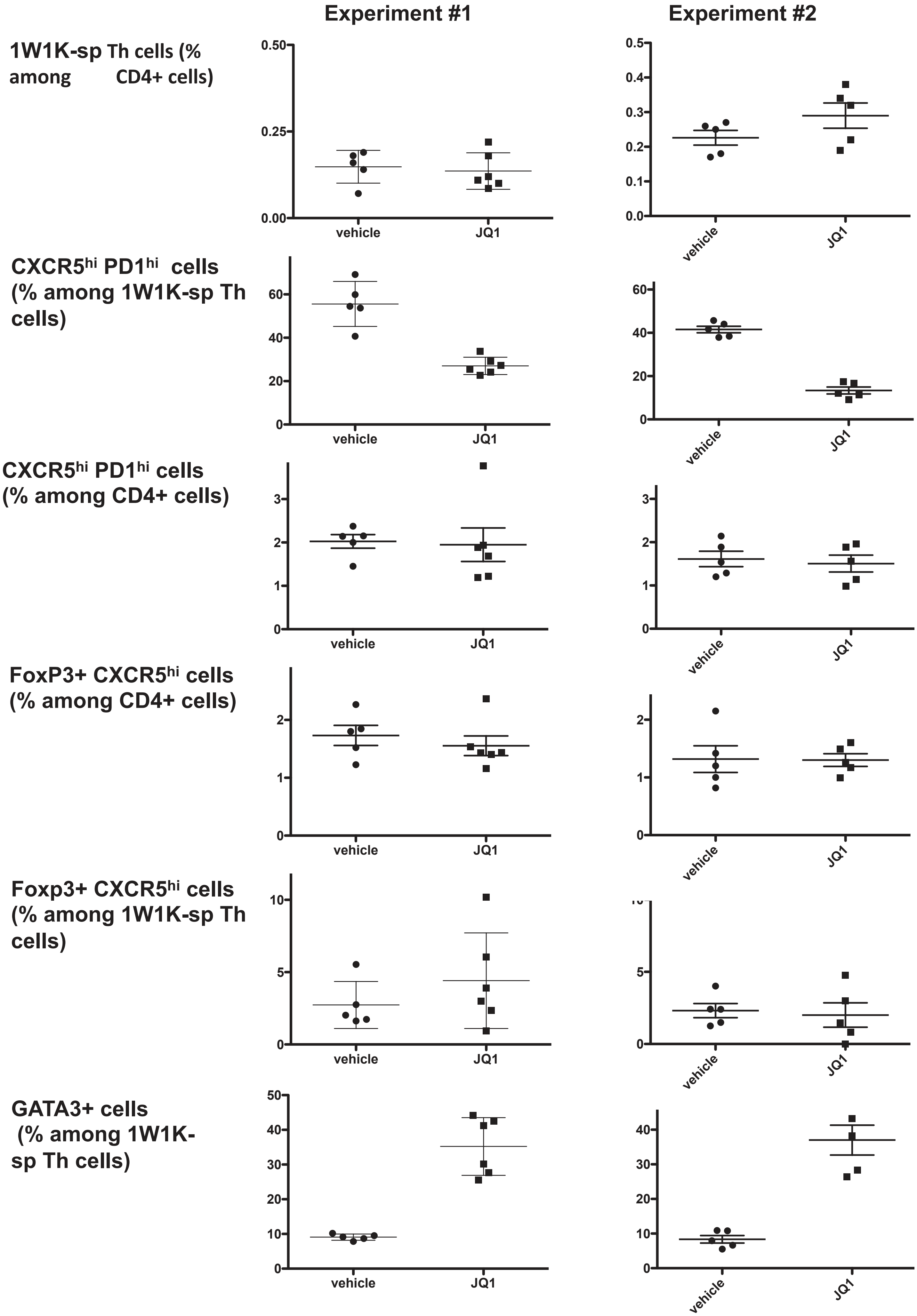

Supplementary figure 5: Replicate experiments for effect of JQ1 on Ag-specific T cells Intraperitoneal and subcutaneous immunisation of C57BL6 mice with 1W1K-OVA in Alum, with or without daily JQ1 treatment from day -1 to day 8 (Exp #1, right: n=5 for vehicle, n=6 for JQ1 / Exp #2, left: n=5 for vehicle, n=5 for JQ1).

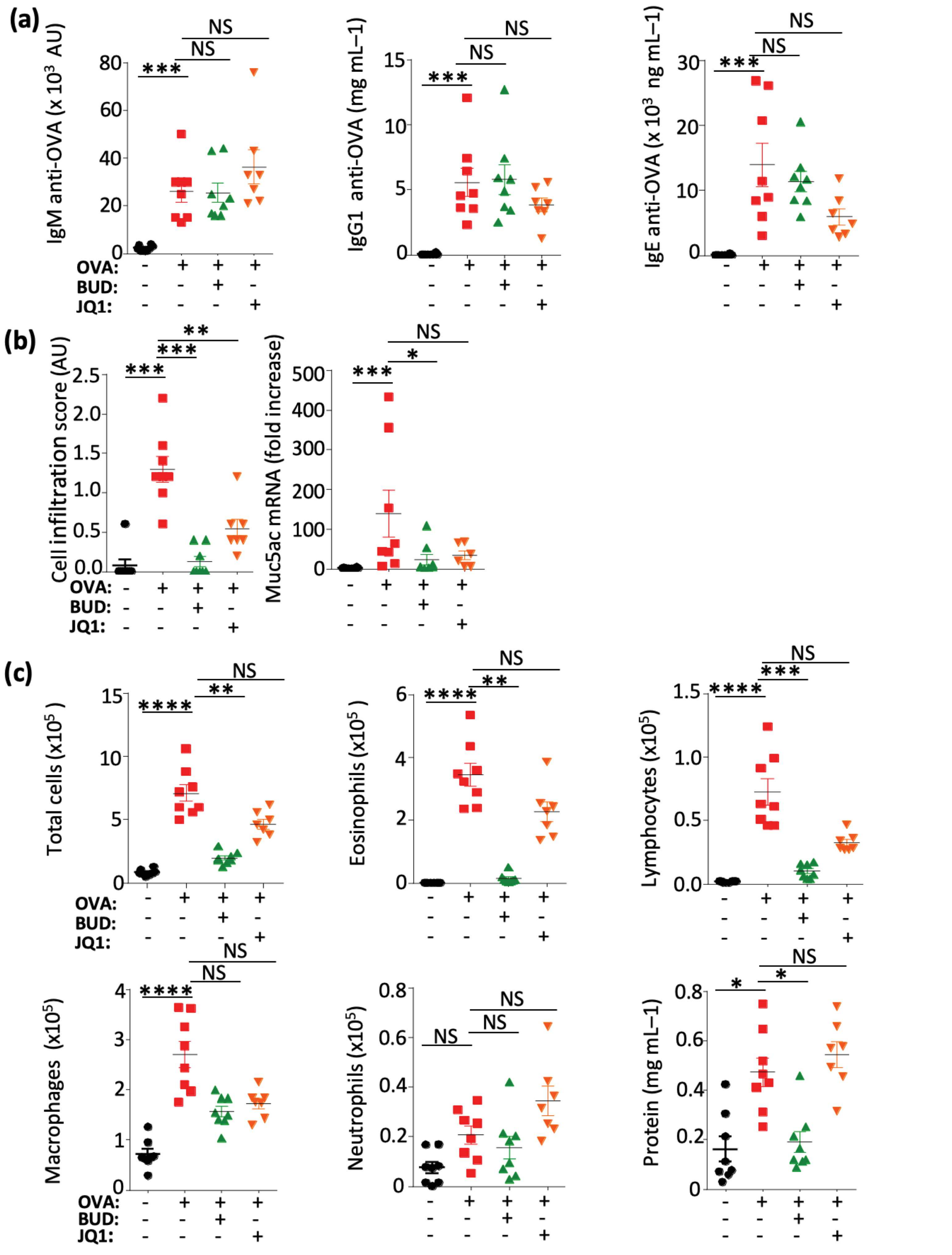

Supplementary figure 6: Replicate experiments for *in vivo* effects of JQ1 on class-switching, Ab production and lymphocyte infiltration in an airway inflammation model. (**a**) Mice administered daily with JQ1 were assayed after repeated rounds of i.p. and i.t. immunization by ovalbumin for levels of Ag-specific IgM, IgG1 and IgE Abs after 25 days. (**b**) Mice administered daily with 50 mg kg^{-1} JQ1 for 4 weeks displayed lower inflammation and less cell infiltration in lungs, together with (**c**) lower counts of lymphocytes and lower protein levels, in broncho-alveolar fluid (BALF). BUD: Budesonide. a-c, values are means ± S.E.M.. Groups include 8 mice per condition. NS: not significant. $*P < 0.05$, $**P < 0.01$, $***P < 0.001$ using non-parametric Kruskal-Wallis followed by Dunn's multiple comparison test.

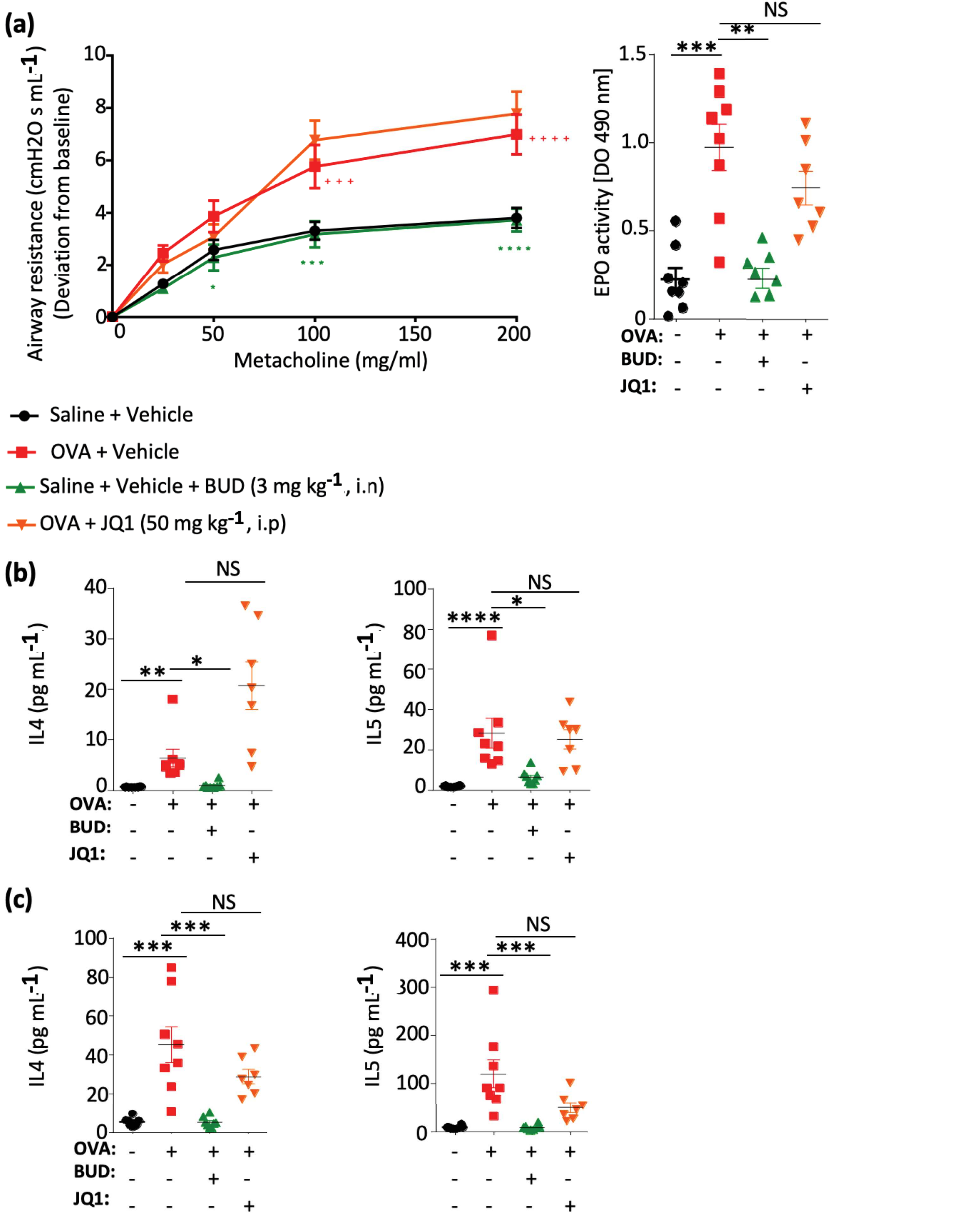

Supplementary figure 7: Replicate experiments for *in vivo* effects of JQ1 on lung function and on Th cytokine production in mice with OVA-induced lung inflammation. (**a**) After repeated i.p. and i.t. OVA administration triggering allergic asthma and compared to those simply receiving OVA only, mice receiving daily steroids (but not those receiving JQ1) showed significantly lower airway resistance (**a, left**), and lower lung eosinophil peroxidase activity (**a, right**). (**b**) Mice immunized and administered daily with steroids, displayed lower levels of IL-4 and IL-5 in broncho-alveolar fluid (BALF), and (**c**) lower levels of IL-4 and IL-5 in lungs, while JQ1 had opposite effects. BUD: Budesonide. Values are means ± s.e.m. Groups include 8 mice per condition tested. NS: not significant. $*P < 0.05$, $**P < 0.01$, $***P < 0.001$ using non-parametric Kruskal-Wallis followed by Dunn's multiple comparison test.
